# miR-502-5p affects gastric cancer progression by targeting PD-L1

**DOI:** 10.1186/s12935-020-01479-2

**Published:** 2020-08-15

**Authors:** Wendao You, Xiaoyu Liu, Yang Yu, Chen Chen, Yujia Xiong, Yiting Liu, Yibin Sun, Chenhuan Tan, Hanshuo Zhang, Yadong Wang, Rui Li

**Affiliations:** 1 Department of Gastroenterology, First Affiliated Hospital of Soochow University, Suzhou, 215006 China; 2 Yulin No.2 Hospital, Yulin, 719000 China; 3 GenoArray Biotech, Suzhou, China; 4 Genex Health Co., Ltd, Beijing, China

**Keywords:** Gastric cancer/carcinoma (GC), miR-502-5p, PD-L1, CD40, STAT3

## Abstract

**Background:**

Studies have shown that miR-502-5p functions as a tumor suppressor and is associated with tumor growth and metastasis. This study intends to uncover the potential mechanism of miR-502-5p functioning as a tumor suppressor in gastric cancer.

**Methods:**

Expression levels of miR-502-5p and PD-L1 were measured by using qRT-PCR. Cell proliferation abilities were examined by EDU incorporation assay. Cell migration, invasion and cell cycle analysis of cells were determined by transwell assay, transwell-matrigel assay and flow cytometry, respectively. The relationship between miR-502-5p expression and the overall survival of xenograft tumor mice was statistically analyzed. Bioinformatics analysis and luciferase reporter assays were applied to analyze the relationship between miR-502-5p and CD40, STAT3 or PD-L1. Expressions of CD40, STAT3 and PD-L1 at protein level were detected by western blot.

**Results:**

The results showed that miR-502-5p was significantly downregulated in gastric cancer tumor tissues compared with adjacent normal tissues. Overexpression of miR-502-5p significantly attenuated the proliferation, migration/invasion and induced the G1 phase arrest of gastric cancer cells. Consistently, miR-502-5p suppressed tumor growth and metastasis in vivo. Mechanically, we demonstrated that miR-502-5p had inhibited the malignant behaviour of gastric cancer by down-regulating PD-L1 expression at transcriptional level and post-transcriptional levels.

**Conclusions:**

These findings suggest that miR-502-5p acts as a tumor suppressor in gastric cancer (GC). MiR-502-5p/PD-L1 may be a novel therapeutic target in GC treatment.

## Background

Gastric cancer (GC) is one of the most common cancers diagnosed in China, which was associated with rather poor survival [1–5]. Despite the diagnosis and treatment have improved during the past decades [6, 7], the prognosis of patients especially with advanced GC is still intractable [8–10]. Therefore, it is essential to explore the underlying molecular mechanism of GC development so as to develop new strategy to improve therapy treatment.

MicroRNAs (miRNAs) are a series of short non-coding RNAs that could negatively regulate genes expression by binding to their 3′ untranslated regions (UTRs) [11, 12]. Their target genes play an important role in multiple biological processes such as cell proliferation, cell cycle control, cell apoptosis, cell migration and invasion. Dysregulation of miRNAs has been reported to be associated with tumorigenesis [13–15]. Studies have showed that several microRNAs are involved in gastric cancer development. For example, microRNA-34a regulates proliferation and apoptosis of gastric cancer by targeting silent information regulator 1 (SIRT1) [16]. Downregulation of miR-491-5p promotes gastric cancer metastasis by inducing EMT via regulation of SNAIL and FGFR4 [17]. MicroRNA-338 inhibits proliferation, migration and invasion of gastric cancer cells by the Wnt/β-catenin signaling pathway [18].

MiR-502-5p is reported to be a tumor suppressor noncoding RNA that is involved in malignant carcinomas. Increased expression of miR-502-5p inhibits cell proliferation and tumor growth in hepatocellular carcinoma [19]. Decreased miR-502-5p expression is significantly associated with poor overall-survival of patients with breast cancer [20]. In the progression of gastric cancer (GC), one study shows that SNP rs56288038 (C/G) in IRF-1 3′UTR promotes GC development by enhancing the regulatory role of miR-502-5p in IRF-1 expression [21]. Interestingly, another study reveales that circDLST promotes the tumorigenesis and metastasis of gastric cancer by sponging miR-502-5p [22]. However, the biological functions and regulatory mechanisms of miR-502-5p in GC are still largely unknown.

Programmed cell death ligand 1 (PD-L1) expression is observed in many malignant tumors and is associated with poor prognosis including gastric cancer (GC). Recent studies have shown that anti-PD-L1/PD-1 antibodies could block tumor progression in patients with non-small-cell lung cancer, melanoma and renal-cell cancer [23–25]. However, it is controversial for the relationship between PD-L1 expression and prognosis in GC. A study reported that patients with PD-L1 positive tumor cells had a significantly improved prognosis [26]. Conversely, another study showed that high PD-L1 expression was a significant adverse prognostic factor [27]. Based on these findings, we speculated that the regulatory mechanism of PD-L1 in gastric cancer is very complex.

In this study, we found that miR-502-5p was decreased in gastric cancer tissues and cell lines. Overexpression of miR-502-5p inhibited gastric cancer cells proliferation and metastasis in vitro and in vivo. Moreover, miR-502-5p overexpression was associated with better prognosis of xenograft tumor mice. Mechanically, these results indicated that miR-502-5p acted as a tumor suppressor by down-regulating PD-L1 expression via inhibiting the CD40/STAT pathway at the transcriptional level and binding to the 3′UTR of PD-L1 mRNA at the post-transcriptional level in gastric cancer. Therefore, miR-502-5p might be applied as a prognosis marker and PD-L1 as a potential therapy target in gastric cancer.

## Materials and methods

### Cell culture and transfection

Human gastric cancer cell lines SGC-7901, BGC823, MGC803 and GES-1 were obtained from the Cell Resource Center, Peking Union Medical College. Cells were cultured at 37 °C in a humidified atmosphere of 5% CO_2_ in DMEM medium (Sigma-Aldrich, UK) supplemented with 10% fetal bovine serum (Gibco, Brisbane, Australia) and 100 U/mL penicillin–streptomycin (Solarbio, Beijing, China). Transfection experiments were performed with vectors, 20 nM siRNA or 20 nM miRNA using Lipofectamine 2000 (Invitrogen, USA) according to manufacturer’s protocol when cells reached 70% confluency.

The sequences of miRNA mimic and siRNA primers were as follows:

has-miR-502-5p mimic, 5′-AUCCUUGCUAUCUGGGUGCUA-3′ (sense)

si-STAT3, GGGACCUGGUGUGAAUUAUTT (sense)

si-CD40, GUGUCAUCUGCUUUCGAAATT (sense)

### Clinical tissue samples

A total of 25 paired GC and adjacent non-tumor gastric tissues were collected at the First Affiliated Hospital of Soochow University (Suzhou, China). This study was approved by the First Affiliated Hospital of Soochow University. The written informed consents were obtained from all participants.

### Reverse transcription and quantitative real-time PCR (qRT-PCR)

Total RNA was extracted from cells with TRIzol^®^ reagent (Invitrogen, USA). MicroRNA was extracted with the microRNA Extraction kit (Tiangen, China). The cDNA was obtained by using the RT reagent kit (TaKaRa, China). PCR was performed with SYBR Green Real-time kit (TaKaRa, China). The primers of miR-502-5p were obtained from RIBOBIO. The item number were ssD089261711 (RP), ssD809231104 (FP), ssD809230412 (RT Primer). The primers for CD40 mRNA are, forward, 5′-TCAGTGCTGTTCTTTGTGCC-3′ and reverse, 5′-TACAGTGCCAGCCTTCTTCA-3′. The primers for STAT3 mRNA are, forward, 5′-AACTCTTGGGACCTGGTGTG-3′ and reverse, 5′-GGCTTAGTGCTCAAGATGGC-3′.

### EdU incorporation

Cells were transfected as experiments designed. After transfection 48 h, cells were stained with EdU solution (Abcam, iFluor 647) for 4 h. Then cells were incubated with reaction mix to label EdU fluorescently. The stained cells were analyzed by the fluorescence microscope.

### CCK-8 assay

Cells were cultured in 24-well plates. The cell proliferation was examined by CCK-8 assay (Beyotime, C0037). Cells were collected at 1, 2, 3, 4 days. The absorbance at 450 nm was measured after incubation with CCK-8 for 2 h. The experiments were repeated three times.

### Wound healing assay

SGC7901/MGC803 cells were transfected as experiments designed. Then they were seeded in the 6-well plate 5 × 10^5^/well so as to form a cell monolayer overnight. The “wound line” was carefully created by a 200 μL sterile plastic tip. After scratching, the detached cells were removed gently with medium. Cells were cultured in DMEM medium containing 2% FBS for about 24 h. Images were taken and the distances were measured. The wound width was calculated as the gap distance at 24 h/gap distance at 0 h. The wound closure equaled to 1/wound width. Each experiment was replicated three times.

### Transwell assay

Transwell migration and invasion assays were performed with Transwell chamber (Millipore, Billerica, USA). For migration assay, SGC7901/MGC803 cells were transfected as experiments designed. After transfection for 48 h, cells (0.8 × 10^5^) were seeded into the upper chamber of the transwell inserts. The lower chamber was contained with DMEM medium. Cells at the bottom of the inserts were fixed and stained with DAPI after 36 h incubation. Random fields were captured at least five and cell numbers were counted for analysis. For invasion assay, transwell inserts were incubated with Matrigel (BD Biosciences, Franklin Lakes, NJ, USA). The next steps were similar to the migration assay as described above.

### Animal model

Nude mice (8–10 weeks old) were purchased from the Laboratory Animal Centre of Soochow University. The animals were housed in a clean facility under the controlled conditions of 22–24 °C, relative humidity 30–50%, and a 12 h-light–dark cycle. All animal procedures were approved by the Research Ethics Committee of Soochow University (Suzhou, China).

### Xenograft tumor model

A total of 1 × 10^7^ SGC7901 cells containing miR-NC-luc/miR-502-5p-luc were injected intraperitoneally into the nude mice with 1 mL PBS [28]. The fluorescent distribution was traced at 4 weeks and 8 weeks after injection. The tumor volume was determined with the caliper after 8 weeks. The expressions of miR-502, CD40, STAT3 and PD-L1 were measured after mice were sacrificed.

### Luciferase reporter assay

The 3′UTR of human CD40/STAT3/PD-L1 was amplified using PCR and cloned into the PGL3-3′UTR vector, respectively. SGC7901 cells in 24-well plates were co-transfected with miR-502-5p mimic or negative control and reporter constructs using Lipofectamine 2000. Cell extracts were prepared 48 h after transfection, and the luciferase activity was measured using the Dual luciferase reporter assay system (Promega).

### Western blot

Proteins were extracted from the whole cell lysates and separated using 12% SDS-PAGE. After transferring to PVDF membranes (Millipore, Billerica, MA, USA), the membranes were incubated with the primary antibody against STAT3 (1:1000; Abcam, Cambridge, USA) and the internal control anti-β-actin (1:2000; Cell Signaling Technology). The membranes were incubated with secondary antibodies (1:5000; Abcam, Cambridge, USA). All bands were detected with the enhanced chemiluminescence (ECL) kit (Thermo Fisher Scientific) and quantified using ImageJ software.

### Statistical analysis

SPSS software (17 version) and GraphPad Prism software (GraphPad Software, La Jolla, CA, USA) were used for statistical analysis. Data were expressed as mean ± standard error of the mean. Statistical significance was determined at P < 0.05.

## Results

### miR-502-5p is decreased in human GC tissues

**Fig. 1:**
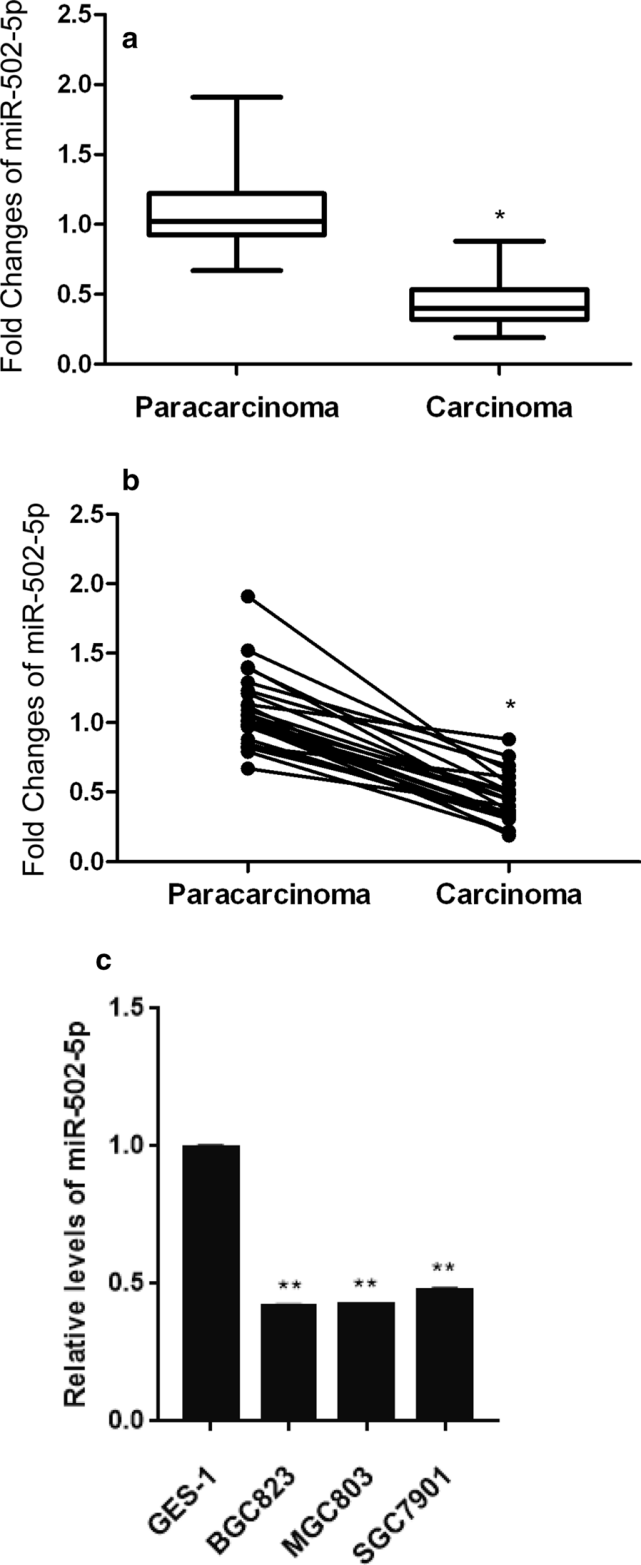
Relative expression of miR-502-5p at mRNA level was evaluated in gastric carcinoma tissues and gastric cancer cell lines by qRT-PCR. **a**, **b** miR-502-5p expression at mRNA level was analyzed in GC tissues (n = 25) and their corresponding nontumorous tissues (n = 25). **c** qRT-PCR was used to measure miR-502-5p expression at mRNA level in GES-1, BGC823, MGC803 and SGC7901. *P < 0.05, **P < 0.01 compared with GES-1

To delineate the role of miR-502-5p in gastric cancer (GC), we collected 25 pairs of GC and its corresponding noncancerous tissues. Firstly we measured the expression of miR-502-5p by qRT-PCR. The relative expression of miR-502-5p was down-regulated in GC tissues (Fig. 1a, b), indicating that miR-502-5p was mainly distributed in corresponding non-tumorous tissues. Moreover, the miR-502-5p expression in the human gastric cell lines (BGC823, MGC803, and SGC7901) was relatively lower compared to GES-1 cells (normal gastric epithelial cells, Fig. 1c, all P < 0.01). Thus, SGC7901 and MGC803 were chosen to be used for later research. These results suggest that downregulation of miR-502-5p is important in GC development.

### miR-502-5p inhibits gastric cancer cell proliferation

**Fig. 2:**
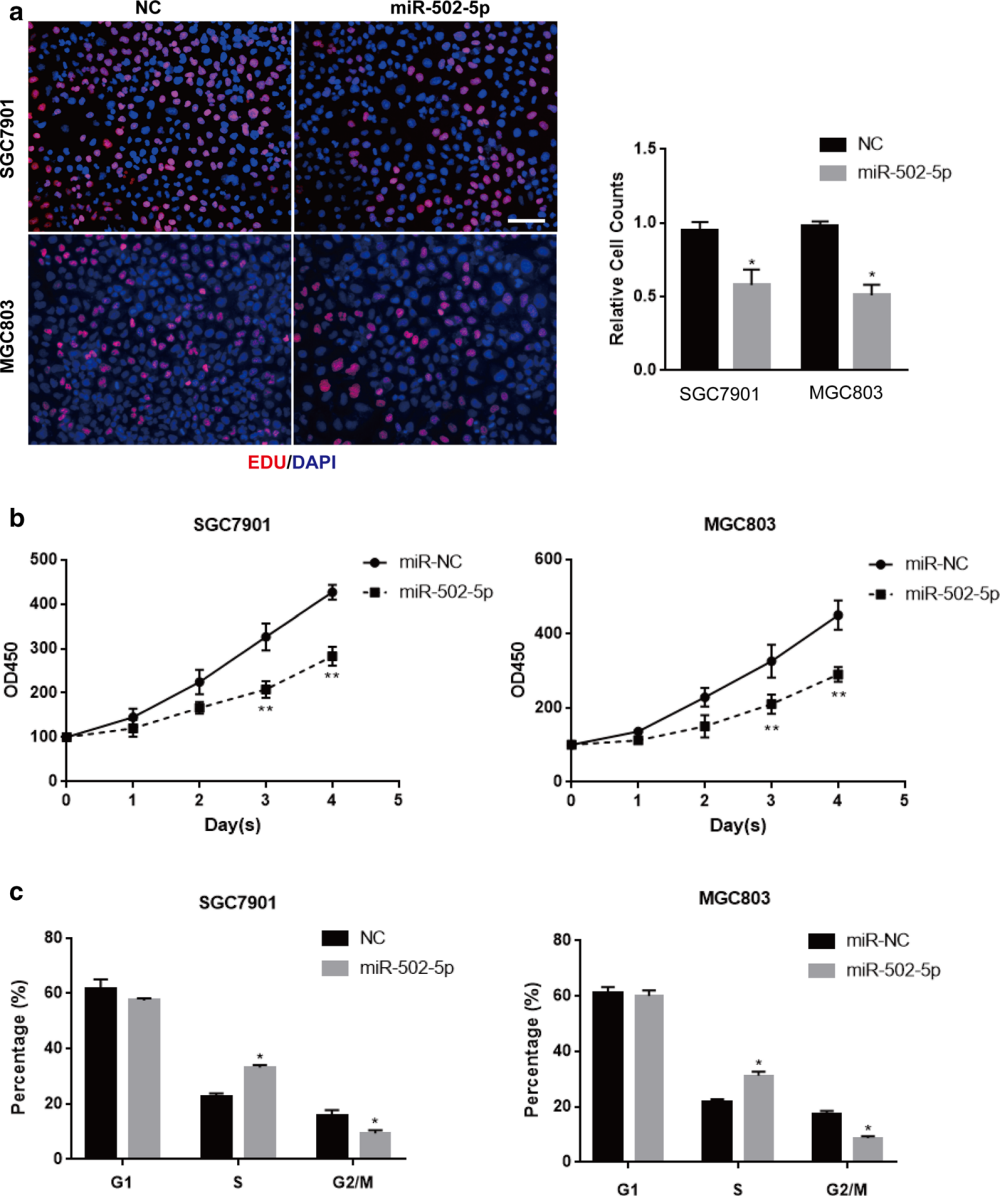
miR-502-5p inhibits gastric cancer cells proliferation in vitro. **a** Proliferous SGC7901 and MGC803 cells transfected with miR-502-5p or miR-NC were examined by EdU immunostaining assays. EdU positive cells were counted and captured. Values are shown as the mean ± s.d in three independent experiments. *P < 0.05, **P < 0.01. **b** The cell proliferation of SGC7901 and MGC803 were measured by drawing cell growth curve at 1, 2, 3 and 4 days. OD450 was obtained at the certain time point. **c** Cell cycle was analyzed by FACS in miR-502-5p-transfected or miR-NC-transfected SGC7901 and MGC803 cells. The overexpression of miR-502-5p induced S phase arrest in GC cells. *P < 0.05, **P < 0.01

To explore the possible role of miR-502-5p in gastric cancer cells, we conducted functional experiments using the SGC7901 and MGC803 cell lines. After transfecting gastric cancer cells (SGC7901 and MGC803) with miR-502-5p mimic or miR-NC, we detected cell proliferation with the EdU incorporation assay. Results showed that the number of proliferative cells was obviously reduced in SGC7901 and MGC803 cells transfected with miR-502-5p mimic compared to the miRNA negative control (P < 0.01) (Fig. 2a). The expression levels of miR-502-5p in the cells were performed by qRT-PCR, as shown in Additional file 1: Figure S1. Additionally, the growth curve indicated that the increased growth rate over time also slowed down when transfected with miR-502-5p in both cell lines (Fig. 2b). To investigate the potential cellular function of the suppressive role of miR-502-5p, we performed cell cycle analyses in gastric cancer cells SGC7901 and MGC803 with overexpression of miR-502-5p mimic or miR-NC. The results demonstrated that cells transfected with miR-502-5p mimic had increased cell numbers in the S phase and decreased cell numbers in G2/M phase for both cell lines, suggesting that miR-502-5p overexpression induced the cell cycle arrest might occur in G1 checkpoints in both SGC7901 and MGC803 cells (Fig. 2c).

### miR-502-5p suppresses migration and invasion of gastric cancer cells

**Fig. 3:**
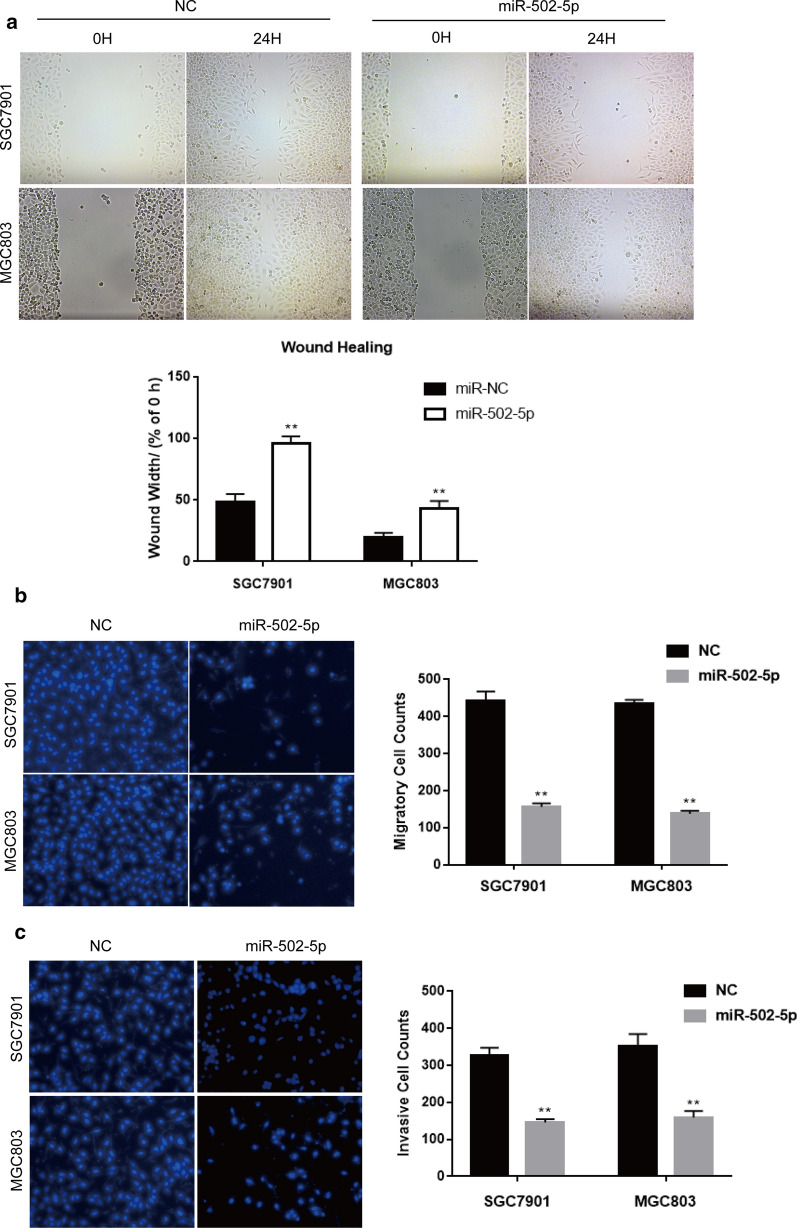
miR-502-5p overexpression suppresses gastric cancer cells migration and invasion. **a** The wound healing analysis showed that miR-502-5p overexpression inhibited the migration ability of gastric cancer cells SGC7901 and MGC803. **P < 0.01, ***P < 0.001. The representative images were shown on top. Magnification ×200. **b** Transwell assays were performed to determine the migration abilities of SGC7901 and MGC803 cells transfected with miR-502-5p or miR-NC. The number of cells migrating from the transwell membrane was quantified after incubation for 24 h. Magnification ×200. Data are representative of three similar experiments. *P < 0.05, **P < 0.01. **c** The invasive ability was investigated by transwell-matrigel assay. The number of cells invading through the transwell membrane coated with matrigel was quantified after incubation for 24 h. Magnification ×200. Data are representative of three similar experiments. *P < 0.05, **P < 0.01

To examine the effects of miR-502-5p overexpression on migration and invasion in gastric cancer cells, we did the wound healing and transwell assays. The results showed that the wound width in miR-502-5p overexpressing gastric cancer cells was significantly greater compared to the negative control, suggesting that miR-502-5p suppressed SGC7901 and MGC803 migration (Fig. 3a). Consistently, the transwell assays results were in line with the wound healing analysis, of which the cell migration of miR-502-5p-expressing gastric cells was inhibited about 50% both in SGC7901 and MGC803 cells (Fig. 3b). The invasive ability was determined by transwell-matrigel analysis. Results showed that increased miR-502-5p expression could inhibit cell invasion to about 44% in SGC7901 and 41% in MGC803 cancer cells (Fig. 3c). Taken together, these findings show that miR-502-5p inhibits proliferative and metastatic traits in gastric cancer cells in vitro.

### miR-502-5p suppresses gastric cancer tumorigenesis and metastasis in vivo

**Fig. 4:**
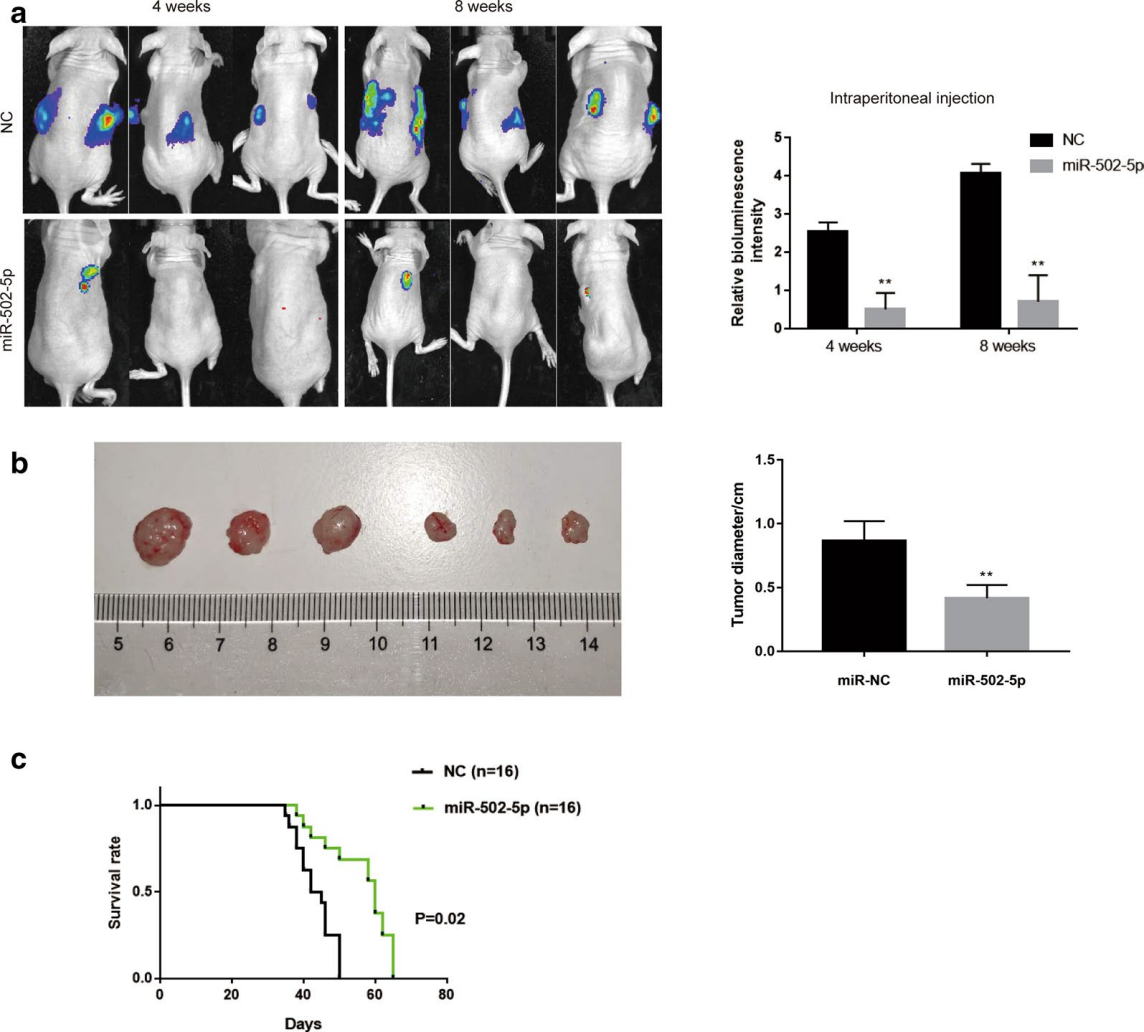
miR-502-5p overexpression inhibited gastric cancer tumorigenesis and metastasis in the abdominal metastatic gastric cancer mice model. **a** Representative bioluminescence images of gastric cancer tumor growth and local metastasis in miR-502-5p and miR-NC group with intraperitoneal injection after 4 weeks and 8 weeks. Tumors were observed by in vivo optical imaging. **b** Tumor size in nude mice was measured with intraperitoneal injection after 8 weeks. **P < 0.01. **c** The overall survival rates in each group were estimated by the Kaplan–Meier method (P = 0.02)

To further evaluate whether miR-502-5p contributed to gastric cancer development, we established abdominal metastatic gastric cancer models. As shown in Fig. 4a, 4 weeks after injection, the group with miR-502-5p overexpression exhibited almost no tumor growth compared to those in the control. Eight weeks after injection, there was a limited tumor growth and no intraperitoneal metastasis with miR-502-5p group whereas the control group exhibited an obvious tumor growth and abdominal diffusion, as demonstrated in the bioluminescence intensity results. In addition, tumor size of mice with miR-502-5p overexpression was significantly less than the control group (Fig. 4b). To further investigate the potential relationship between miR-502-5p expression and tumor mice survival, Kaplan–Meier analysis was used to evaluate the effects of miR-502-5p expression on overall survival. The results indicated that mice bearing miR-NC-luc tumors exhibited a significantly poor survival than miR-502-5p-injected group (Fig. 4c, P = 0.02). The results suggest that miR-502-5p strongly inhibits tumor growth and metastasis, indicating that miR-502-5p plays a key role in gastric cancer progression and development.

### miR-502-5p targets PD-L1 via CD40/STAT3 pathway

**Fig. 5:**
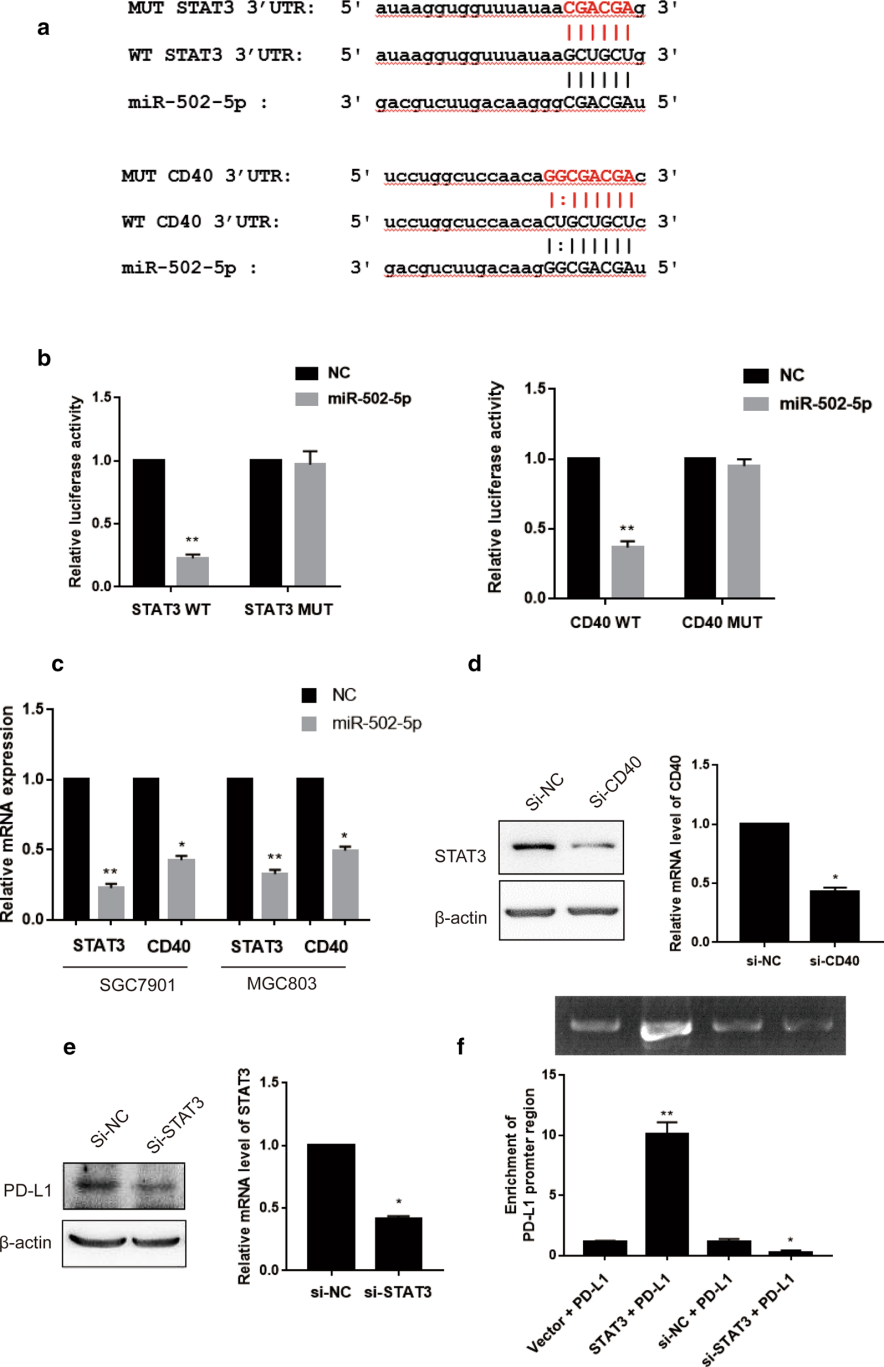
miR-502-5p targets PD-L1 via CD40/STAT3 pathway. **a** Bioinformatics predication of interaction between miR-502-5p and CD40 or STAT3. **b** Luciferase reporter assay showed that miR-502-5p overexpression significantly repressed the luciferase activity of CD40-wt and STAT3-wt, while the luciferase activities of CD40-mut and STAT3-mut were not affected in SGC7901 cells. **c** The expressions of CD40 and STAT3 determined by qRT-PCR in two gastric cancer cell lines were significantly decreased following miR-502-5p transfection. **d**, **e** After transfection with siRNA against CD40 (si-CD40) or siRNA against STAT3 (si-STAT3) for 24 h, total cellular lysates were collected and subjected to the western blot analysis with indicated antibodies. The knockdown efficiency of si-CD40 or si-STAT3 was determined by qRT-PCR. **f** Chromatin immunoprecipitation (ChIP) assay was performed in SGC7901 cells using the STAT3 antibody. The IgG antibody served as a negative control. Analysis was conducted using specific primers for the promoter region of PD-L1

Based on the prediction by bioinformatics, we selected some highly predicted target genes that were significantly regulated by miR-502-5p. We constructed the 3′ untranslated regions (3′UTRs) of these genes conjugated with luciferase plasmid for reporter assays. We observed that the 3′ UTR of CD40 and STAT3 mRNA harbored sequences complementary to the miR-502-5p as shown in Fig. 5a. We wondered whether the binding site was specific, then we constructed the mutant CD40/STAT3 3′-UTR plasmids. The luciferase reporter assays demonstrated that miR-502-5p remarkably inhibited the luciferase activity in SGC7901 cells transfected with wild-type CD40/STAT3 reporter but not in the mutant reporters (Fig. 5b), indicating that miR-502-5p directly regulated the 3′-UTR of CD40/STAT3 mRNA. To confirm these results, we performed validation experiments. The expressions of CD40 and STAT3 were decreased with the transient transfection of miR-502-5p in two gastric cancer cell lines by qRT-PCR and western blot (Fig. 5c and Additional file 2: Figure S2). Consistently, the expressions of CD40 and STAT3 were also decreased in miR-502-5p injected mice tumor as shown in Additional file 3: Figure S6. As CD40 being a member of the TNF-receptor essential in activating a broad variety of immune and inflammatory responses, we assumed that the signal transducer and activator of transcription 3 (STAT3) was regulated by CD40. We conducted small interfering RNA (siRNA)-based silencing of CD40. The knockdown efficiency of CD40 was assessed in the meantime. As expected, siRNA-mediated knockdown of CD40 resulted in a significant decrease in STAT3 expression (Fig. 5d). These results indicate that STAT3 is activated by CD40, both of which are inhibited by miR-502-5p overexpression. Recent studies reported that PD-L1 was a downstream target of STAT3 signaling. We quantified PD-L1 expression in STAT3-silenced SGC7901 cells and observed that reduced expression of PD-L1 was in accordance with the knockdown of STAT3 (Fig. 5e). Furthermore, we examined the enrichment of PD-L1 promoter sequence combined with STAT3 by Chromatin IP and observed that the enrichment is relatively more with STAT3 antibody than IgG control (Fig. 5f). Taken together, miR-502-5p-overexpression-regulated CD40/STAT3 dependent signaling pathway can repress PD-L1 expression at the transcriptional level.

### MiR-502-5p regulates PD-L1 expression at the post-transcriptional level

**Fig. 6:**
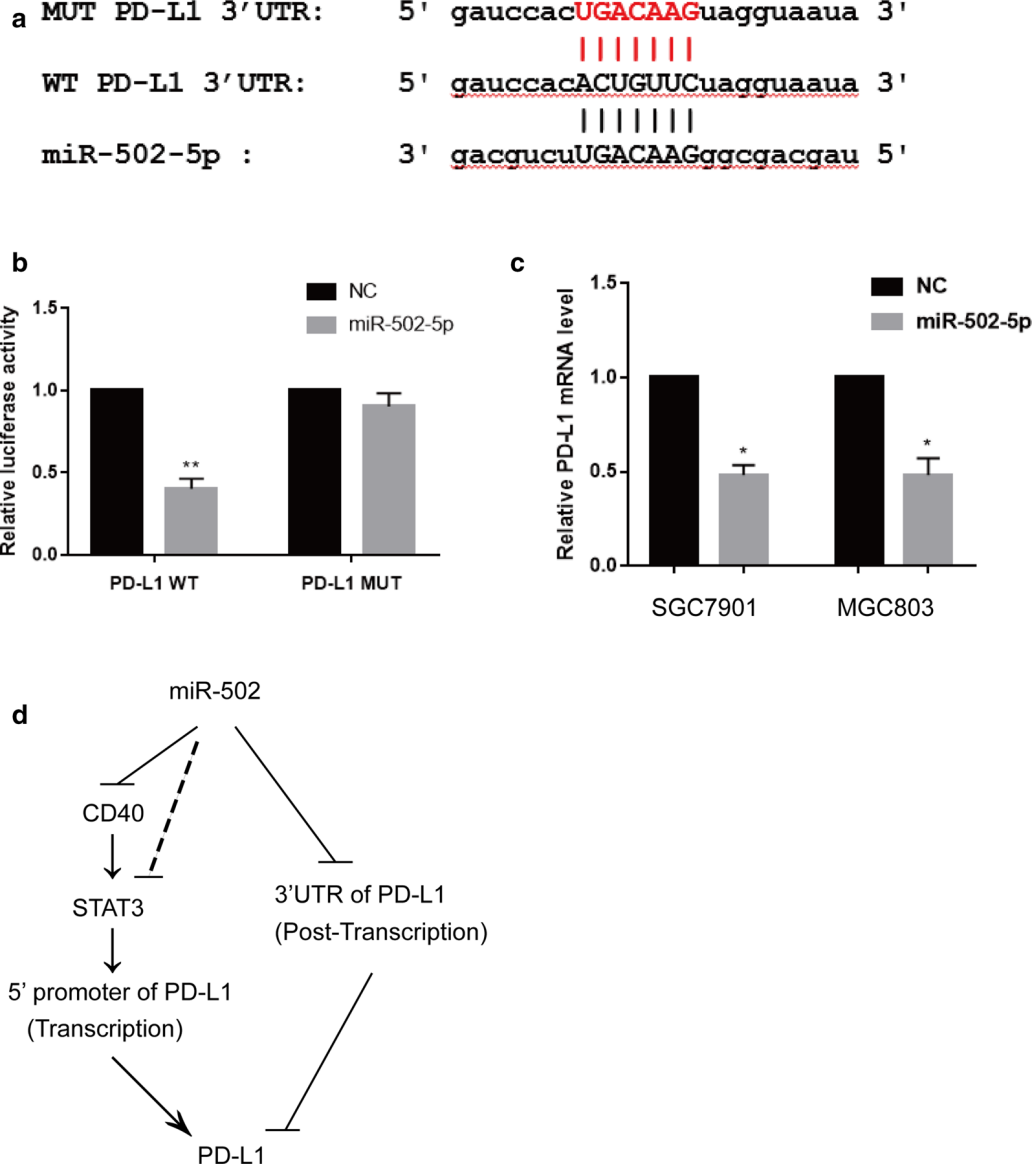
miR-502-5p directly binds to the 3′ UTR region of PD-L1. **a** Bioinformatics predicated binding sequence between miR-502-5p and PD-L1. **b** Luciferase reporter assay showed that miR-502-5p overexpression significantly repressed the luciferase activity of PD-L1-wt, while the luciferase activity of PD-L1-mut was not affected in SGC7901 cells. **c** The expression of PD-L1 in two gastric cancer cell lines was significantly decreased following miR-502-5p transfection determined by qRT-PCR. **d** miR-502-5p induced PD-L1 expression was regulated at both the transcriptional and post-transcriptional levels. A positive regulatory loop was formed by miR-502-5p-CD40/STAT3-PD-L1 in gastric cancer

In view of the results, we wondered whether miR-502-5p directly targeted PD-L1. To verify the hypothesis, we cloned the predicted binding sequence of PD-L1’s 3′UTR into the luciferase reporter plasmid to explore the miR-502-5p’s targets specificity. The results were shown in Fig. 6a, b. The relative luciferase activity of the wild-type 3′-UTR was significantly repressed following miR-502-5p mimic transfection compared to control in SGC7901 whereas the mutant not. Consistently, the PD-L1 mRNA expression was down-regulated both in SGC7901 and MGC803 gastric cancer cell lines transfected with miR-502-5p quantified by qRT-PCR and western blot (Fig. 6c and Additional file 2: Figure S2). In addition, PD-L1 overexpression could restore the miR-502-5p-overexpression induced cellular effects whereas CD40/STAT3 overexpression could partially restore the miR-502-5p-overexpression suppressed gastric cancer cells proliferation, migration and invasion (Additional file 4: Figure S3, Additional file 5: Figure S4 and Additional file 6: Figure S5). To sum up, these results indicate that miR-502-5p regulates PD-L1 expression at the post-transcriptional level (Fig. 6d).

### miR-502-5p is inversely correlated with PD-L1 in gastric cancer tissues

**Fig. 7:**
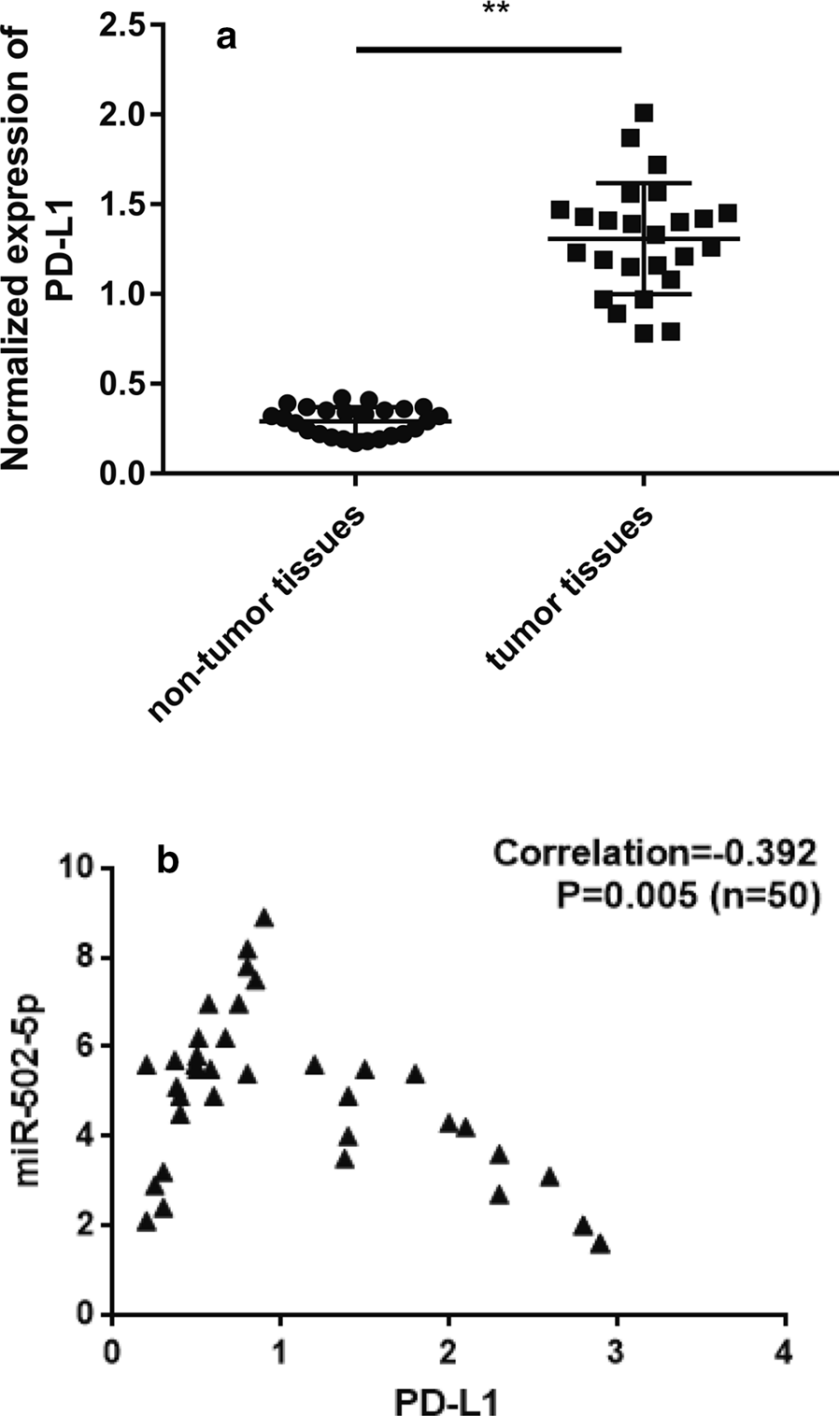
miR-502-5p is inversely correlated with PD-L1. **a** The expression level of PD-L1 was up-regulated in gastric cancer samples (n = 25), as determined by qRT-PCR. **b** Correlation analysis revealed that there was a negative correlation between miR-502-5p and PD-L1 mRNA levels (R = − 0.392, P = 0.005)

The expression of miR-502-5p was significantly down-regulated in gastric cancer tissues compared to adjacent non-tumor tissues (Fig. 1a, b), while the expression level of PD-L1 was obviously higher in gastric cancer tissues than in normal adjacent tissues (Fig. 7a, P < 0.01). Consistently, knockdown of PD-L1 led to suppressed gastric cancer cells proliferation, migration and invasion (Additional file 7: Figure S7). Furthermore, correlation analysis revealed that miR-502-5p expression was inversely correlated with PD-L1 mRNA level in gastric cancer tissues (Fig. 7b, P < 0.05).

## Discussion

Gastric cancer is one of the most common malignant tumors with high morbidity and mortality worldwide [29–31]. It is urgent to enhance the diagnosis and therapy. The increasing understanding of molecular pathways is the basis for innovative therapies. In our study, we focused on the regulation of miR-502-5p in GC Results indicate that miR-502-5p is down-regulated in the human gastric cancer tissues and gastric cancer cells. Overexpression of miR-502-5p could inhibit gastric cancer tumor growth, migration and metastasis in vitro and in vivo, suggesting that miR-502-5p acts as a tumor suppressor role in gastric cancer.

Nowadays, dysregulation of microRNAs play a vital role in GC tumorigenesis [32, 33]. It has been reported that miR-502-5p, as a tumor suppressor microRNA is involved in the development of various cancers. For example, miR-502-5p has been found to be down-regulated in hepatocellular carcinoma [34]. MicroRNA-502-5p is associated with clear cell renal cell carcinoma by mediating histone methyltransferase SET8 expression [35]. These findings prompted us to explore the regulatory mechanism of miR-502-5p in GC. As we all known, microRNAs are involved in tumorigenesis by regulating the expression of target genes.

In our study, we found that CD40, STAT3 and PD-L1 are the targets of miR-502-5p in gastric cancer cells by bioinformatics analysis and luciferase reporter assay. On one hand, STAT3 could regulate PD-L1 expression by binding to its promoter region. MiR-502-5p indirectly inhibiting PD-L1 expression by downregulating CD40 and STAT3 expression, which was at the post-transcription level. On the other hand, miR-502-5p could inhibit PD-L1 expression by binding to its mRNA 3′UTR, which was at the transcription level. Thus, miR-502-5p likely elicits tumor suppressive effects through the dual regulation of PD-L1 expression.

It is known that PD-L1 plays an important role in tumor development. PD-L1 has been speculated to play a major role in suppressing the immune system that allows immune escape of tumor cells [36–40]. Multiple researches show that PD-L1 is correlated with the progression of gastric cancer [40]. For example, PD-L1 is highly expressed in gastric cancer tissues and associated with a poor prognosis in patients with gastric cancer [36, 41]. Overexpression of PD-L1 in peripheral blood may offer an immunological predictor of tumor progression and disease outcome in patients with gastric cancer [42]. What’s more, PD-L1 expression correlated significantly with depth of tumor invasion, distant metastasis, and stage [42]. These studies are in accordance with our findings, that is, PD-L1 was upregulated in GC tumor tissues compared to normal stomach tissues and there was a negative correlation between miR-502-5p and PD-L1 in gastric cancer tissues. The novel mechanism that miR-502-5p regulates PD-L1 expression in two ways may shed light on the immune checkpoint inhibitor therapy in gastric cancer.

Although our findings have provided new insights into the regulatory mechanism of miR-502-5p in GC, studies about the function of miR-502-5p in gastric cancer still remains complicated. For example, Wang et al. found that miR-502-5p may have a potential transcriptional suppressive effect on IRF-1 with *C/G SNP in rs56288038* in gastric cancer, which might have a facilitating effect on GC development [21]. It is interesting to suppose that there are two sides of miR-502-5p in GC. Therefore further studies are required to investigate the therapeutic and prognostic value of miR-502-5p in GC in the future.

## Conclusions

In summary, miR-502-5p acts as a tumor suppressor microRNA that represses gastric cancer tumorigenesis via regulating PD-L1 expression at transcriptional level and post-transcriptional level. Our study indicates that miR-502-5p might be considered as a prognosis marker of gastric cancer and PD-L1 acts as a potential therapeutic target in the management of gastric cancer.

## Supplementary information

**Additional file 1: Figure S1.** Expression of miR-502-5p is determined by qRT-PCR. ***P < 0.001.

**Additional file 2: Figure S2.** Expression of CD40/STAT3/PD-L1 at protein level in miR-502-5p mimic -transfected SGC7901 is measured by western blot.

**Additional file 3: Figure S6.** Expression of the related factors changes in mice tumor tissues. (A) qRT-PCR was performed to measure the expression of CD40, STAT3 and PD-L1 in mice tumor tissues after sacrificed. **P < 0.01. (B) qRT-PCR was performed to measure the expression of miR-502-5p in mice tumor tissues after sacrificed. ***P < 0.001.

**Additional file 4: Figure S3.** PD-L1 overexpression restores the miR-502-5p-overexpression induced cellular effects. (A) CCK8 assays of SGC7901 and MGC803 cells were performed after transfected (un-transfected) with miR-502-5p and miR-502-5p + PD-L1. (B) Wound healing analysis of gastric cancer cells migration was performed after treatment with miR-502-5p or miR-502-5p + PD-L1 or control in SGC7901 and MGC803 cells. Assays were performed in triplicate. (C) Transwell analysis of gastric cancer cells migration and invasion was performed after treatment with miR-502-5p or miR-502-5p + PD-L1 or control in SGC7901 and MGC803 cells. Assays were performed in triplicate. *P < 0.05, ** P < 0.01.

**Additional file 5: Figure S4.** STAT3 overexpression partially restores the miR-502-5p-overexpression induced cellular effects. (A) CCK8 assays of SGC7901 and MGC803 cells were performed after transfected (un-transfected) with miR-502-5p and miR-502-5p + STAT3. (B) Wound healing analysis of gastric cancer cells migration was performed after treatment with miR-502-5p or miR-502-5p + STAT3 or control in SGC7901 and MGC803 cells. Assays were performed in triplicate. (C) Transwell analysis of gastric cancer cells migration and invasion was performed after treatment with miR-502-5p or miR-502-5p + STAT3 or control in SGC7901 and MGC803 cells. Assays were performed in triplicate. *P < 0.05, **P < 0.01.

**Additional file 6: Figure S5.** CD40 overexpression partially restores the miR-502-overexpression induced cellular effects. (A) CCK8 assays of SGC7901 and MGC803 cells were performed after transfected (un-transfected) with miR-502-5p and miR-502-5p + CD40. (B) Wound healing analysis of gastric cancer cells migration was performed after treatment with miR-502-5p or miR-502-5p + CD40 or control in SGC7901 and MGC803 cells. Assays were performed in triplicate. (C) Transwell analysis of gastric cancer cells migration and invasion was performed after treatment with miR-502-5p or miR-502-5p + CD40 or control in SGC7901 and MGC803 cells. Assays were performed in triplicate. *P < 0.05, **P < 0.01.

**Additional file 7: Figure S7.** PD-L1 knockdown inhibits gastric cancer cells proliferation, migration and invasion. (A) The knockdown efficiency of si-PD-L1 was examined by qRT-PCR. (B and C) CCK8 assays of SGC7901 and MGC803 cells were performed after transfected with si-NC or si-PD-L1. (D and E) Transwell analysis of gastric cancer cells was performed after transfected with si-NC or si-PD-L1 in SGC7901 and MGC803 cells. Assays were performed in triplicate. **P < 0.01.

## Data Availability

The datasets supporting the conclusions of this article are included within the article.

## Abbreviations

3′-UTR: 3′ Untranslated regions
PD-L1: Programmed death-ligand 1
GC: Gastric cancer/carcinoma
EdU: 5-Ethynyl-2′-deoxyuridine
CCK-8: Cell Counting Kit-8
qRT-PCR: Reverse transcription and quantitative real-time PCR
