# Supplementary figures and images for: miR-502-5p affects gastric cancer progression by targeting PD-L1
Source: Cancer Cell Int. 2020 Aug 15;20:395. doi: 10.1186/s12935-020-01479-2 (PMC7429713; doi:10.1186/s12935-020-01479-2)

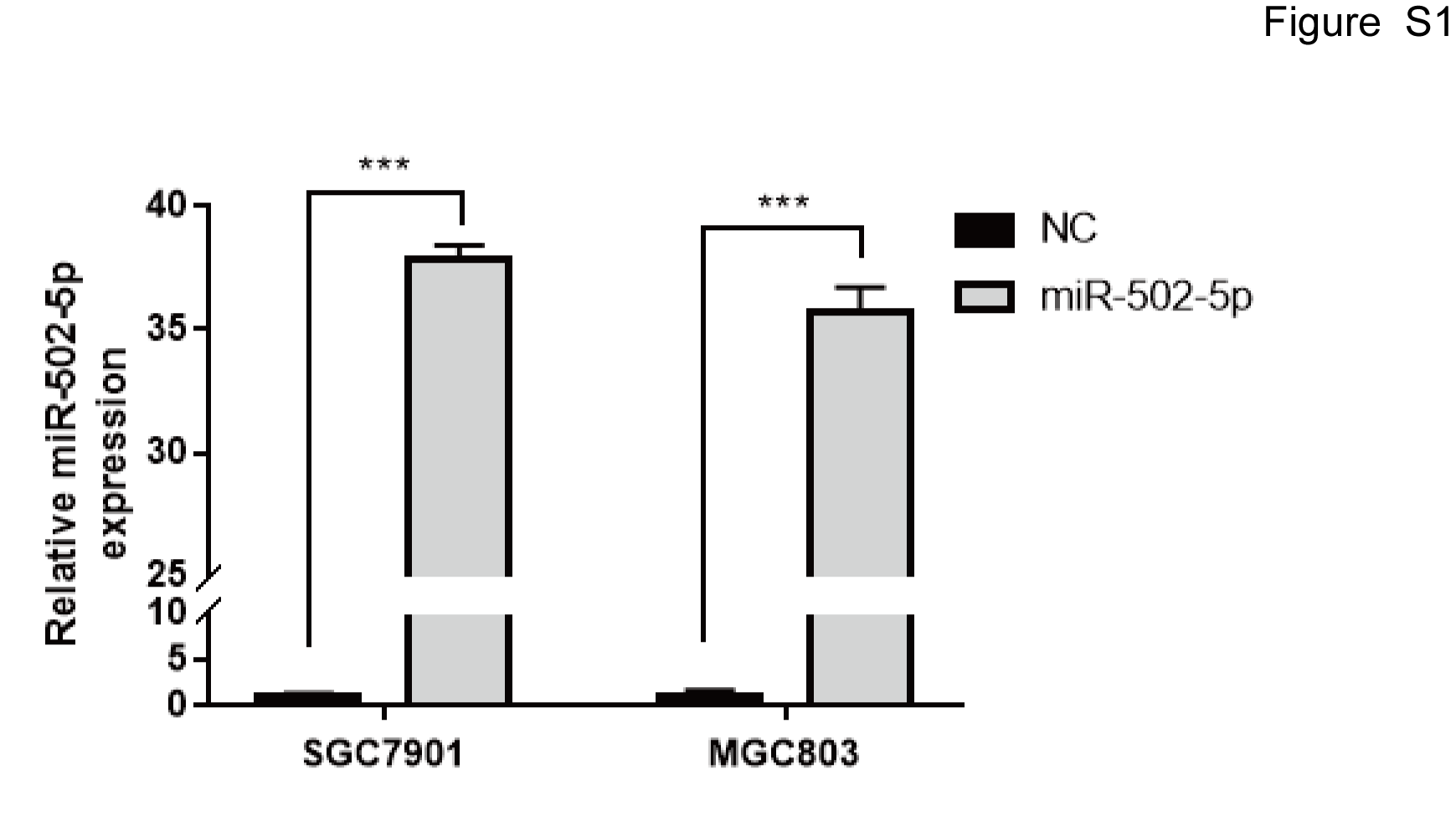

Supplement: Supplementary file 1 — Additional file 1: Figure S1. Expression of miR-502-5p is determined by qRT-PCR. ***P < 0.001. [file 12935_2020_1479_MOESM1_ESM.tif]

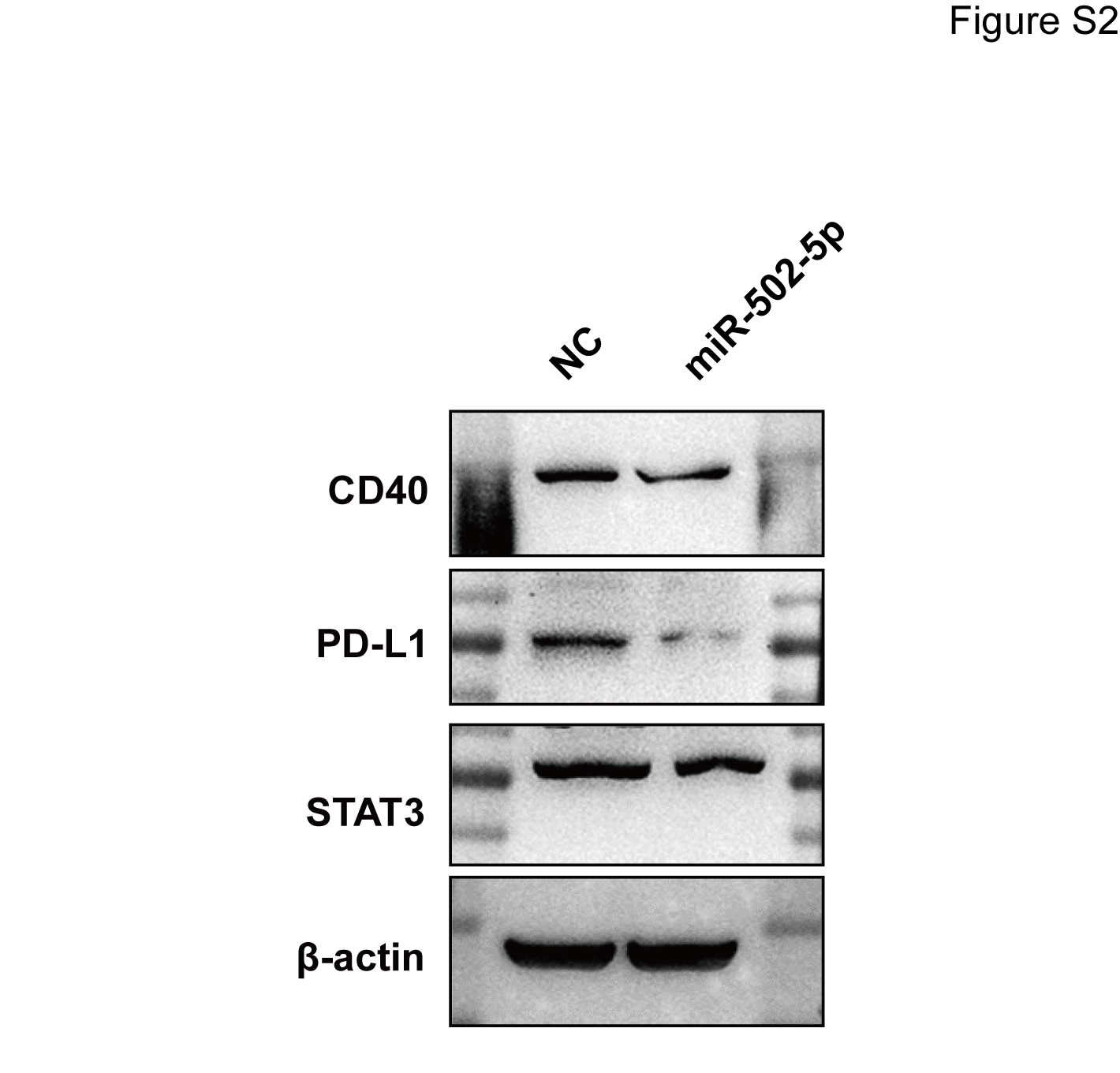

Supplement: Supplementary file 2 — Additional file 2: Figure S2. Expression of CD40/STAT3/PD-L1 at protein level in miR-502-5p mimic -transfected SGC7901 is measured by western blot. [file 12935_2020_1479_MOESM2_ESM.tif]

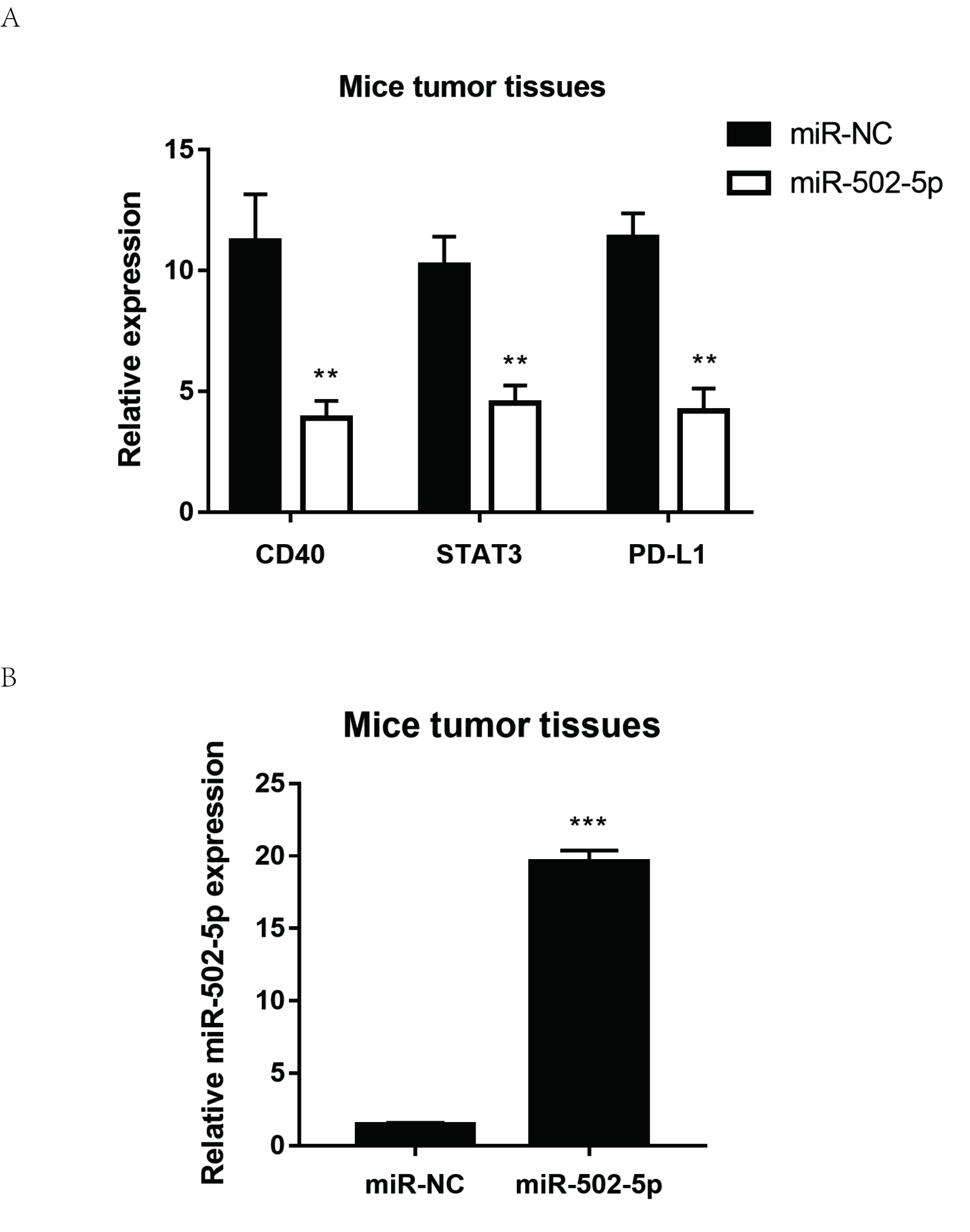

Supplement: Supplementary file 3 — Additional file 3: Figure S6. Expression of the related factors changes in mice tumor tissues. (A) qRT-PCR was performed to measure the expression of CD40, STAT3 and PD-L1 in mice tumor tissues after sacrificed. **P < 0.01. (B) qRT-PCR was performed to measure the expression of miR-502-5p in mice tumor tissues after sacrificed. ***P < 0.001. [file 12935_2020_1479_MOESM3_ESM.tif]

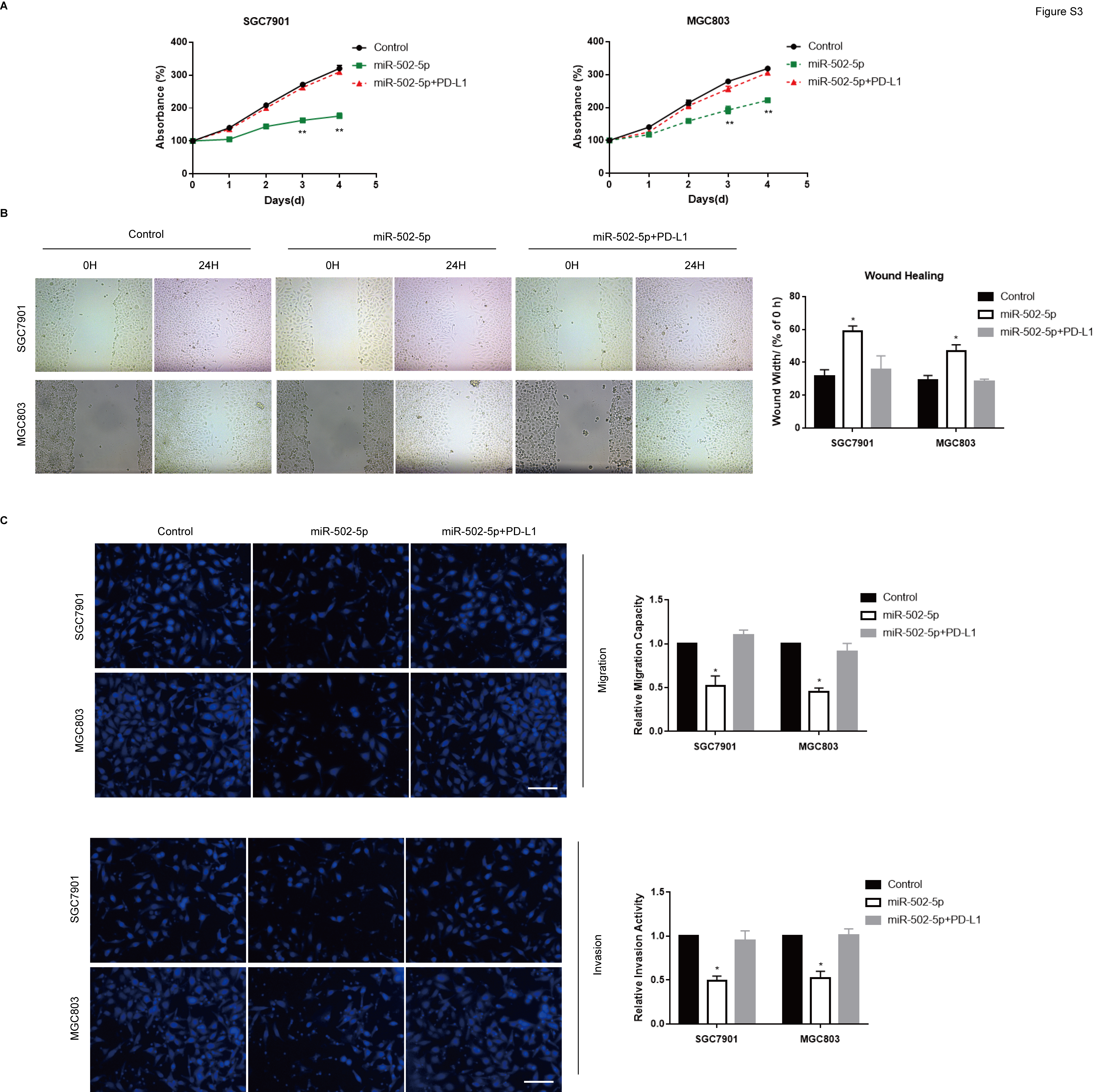

Supplement: Supplementary file 4 — Additional file 4: Figure S3. PD-L1 overexpression restores the miR-502-5p-overexpression induced cellular effects. (A) CCK8 assays of SGC7901 and MGC803 cells were performed after transfected (un-transfected) with miR-502-5p and miR-502-5p + PD-L1. (B) Wound healing analysis of gastric cancer cells migration was performed after treatment with miR-502-5p or miR-502-5p + PD-L1 or control in SGC7901 and MGC803 cells. Assays were performed in triplicate. (C) Transwell analysis of gastric cancer cells migration and invasion was performed after treatment with miR-502-5p or miR-502-5p + PD-L1 or control in SGC7901 and MGC803 cells. Assays were performed in triplicate. *P < 0.05, ** P < 0.01. [file 12935_2020_1479_MOESM4_ESM.tif]

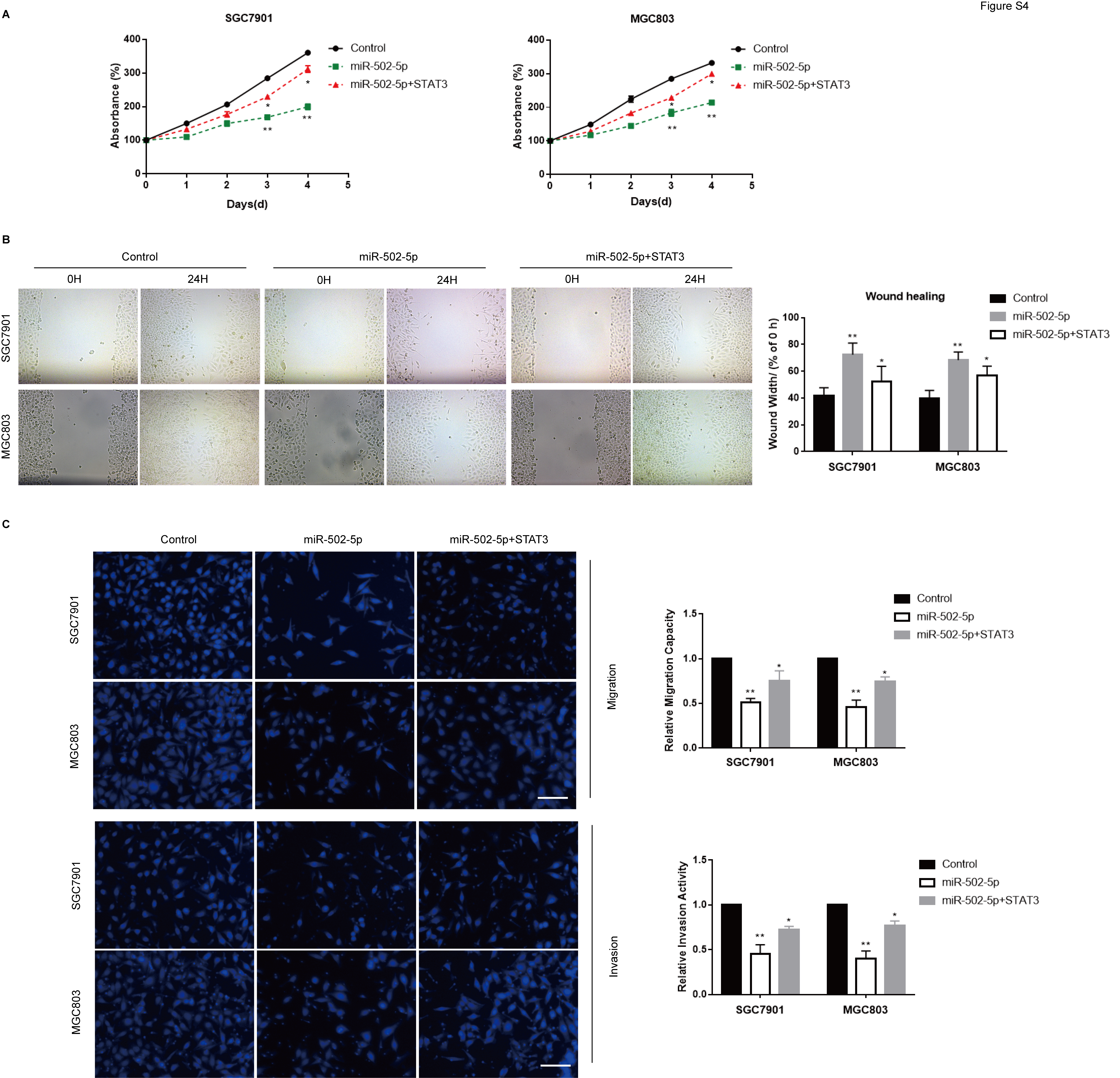

Supplement: Supplementary file 5 — Additional file 5: Figure S4. STAT3 overexpression partially restores the miR-502-5p-overexpression induced cellular effects. (A) CCK8 assays of SGC7901 and MGC803 cells were performed after transfected (un-transfected) with miR-502-5p and miR-502-5p + STAT3. (B) Wound healing analysis of gastric cancer cells migration was performed after treatment with miR-502-5p or miR-502-5p + STAT3 or control in SGC7901 and MGC803 cells. Assays were performed in triplicate. (C) Transwell analysis of gastric cancer cells migration and invasion was performed after treatment with miR-502-5p or miR-502-5p + STAT3 or control in SGC7901 and MGC803 cells. Assays were performed in triplicate. *P < 0.05, **P < 0.01. [file 12935_2020_1479_MOESM5_ESM.tif]

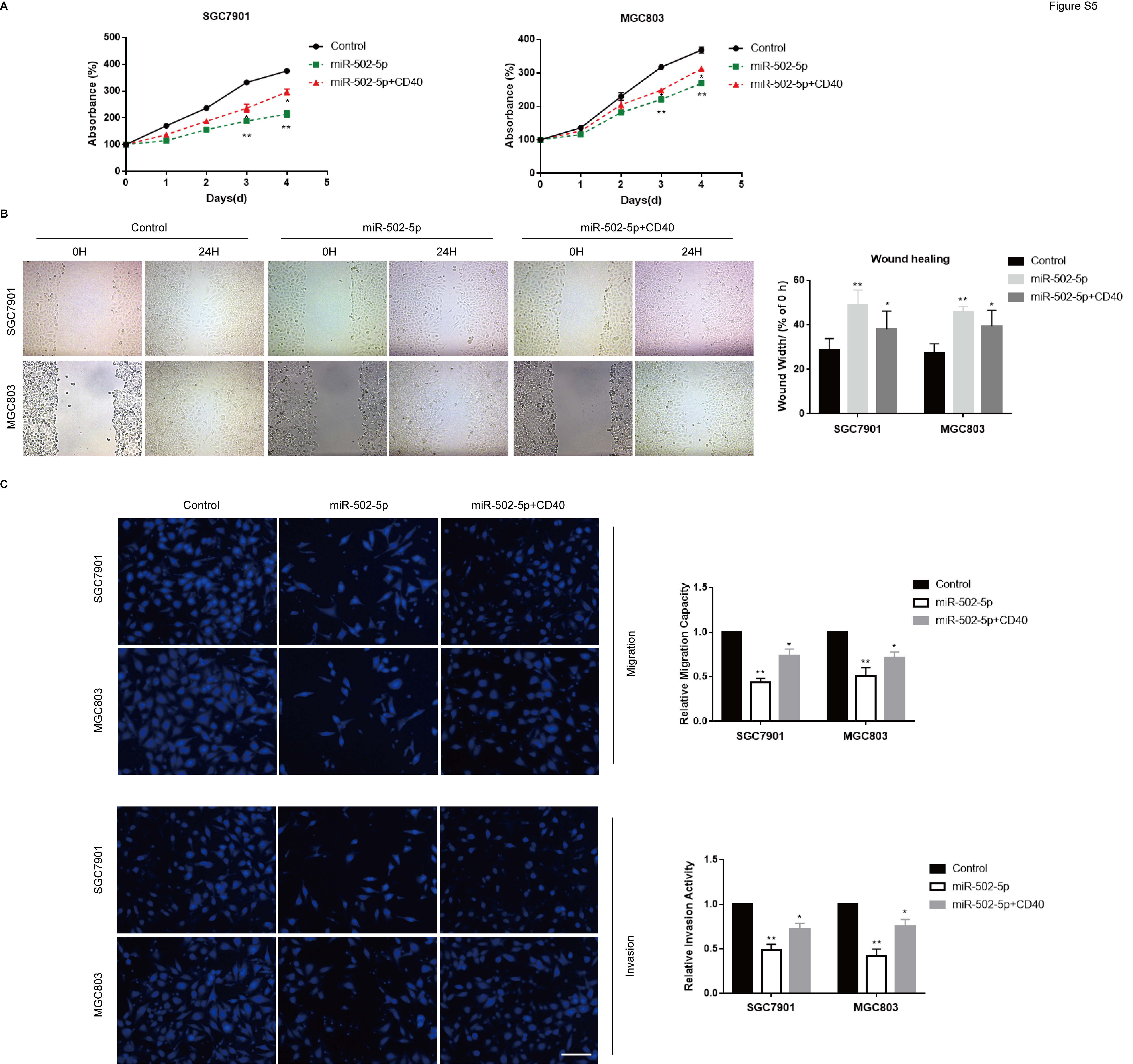

Supplement: Supplementary file 6 — Additional file 6: Figure S5. CD40 overexpression partially restores the miR-502-overexpression induced cellular effects. (A) CCK8 assays of SGC7901 and MGC803 cells were performed after transfected (un-transfected) with miR-502-5p and miR-502-5p + CD40. (B) Wound healing analysis of gastric cancer cells migration was performed after treatment with miR-502-5p or miR-502-5p + CD40 or control in SGC7901 and MGC803 cells. Assays were performed in triplicate. (C) Transwell analysis of gastric cancer cells migration and invasion was performed after treatment with miR-502-5p or miR-502-5p + CD40 or control in SGC7901 and MGC803 cells. Assays were performed in triplicate. *P < 0.05, **P < 0.01. [file 12935_2020_1479_MOESM6_ESM.tif]

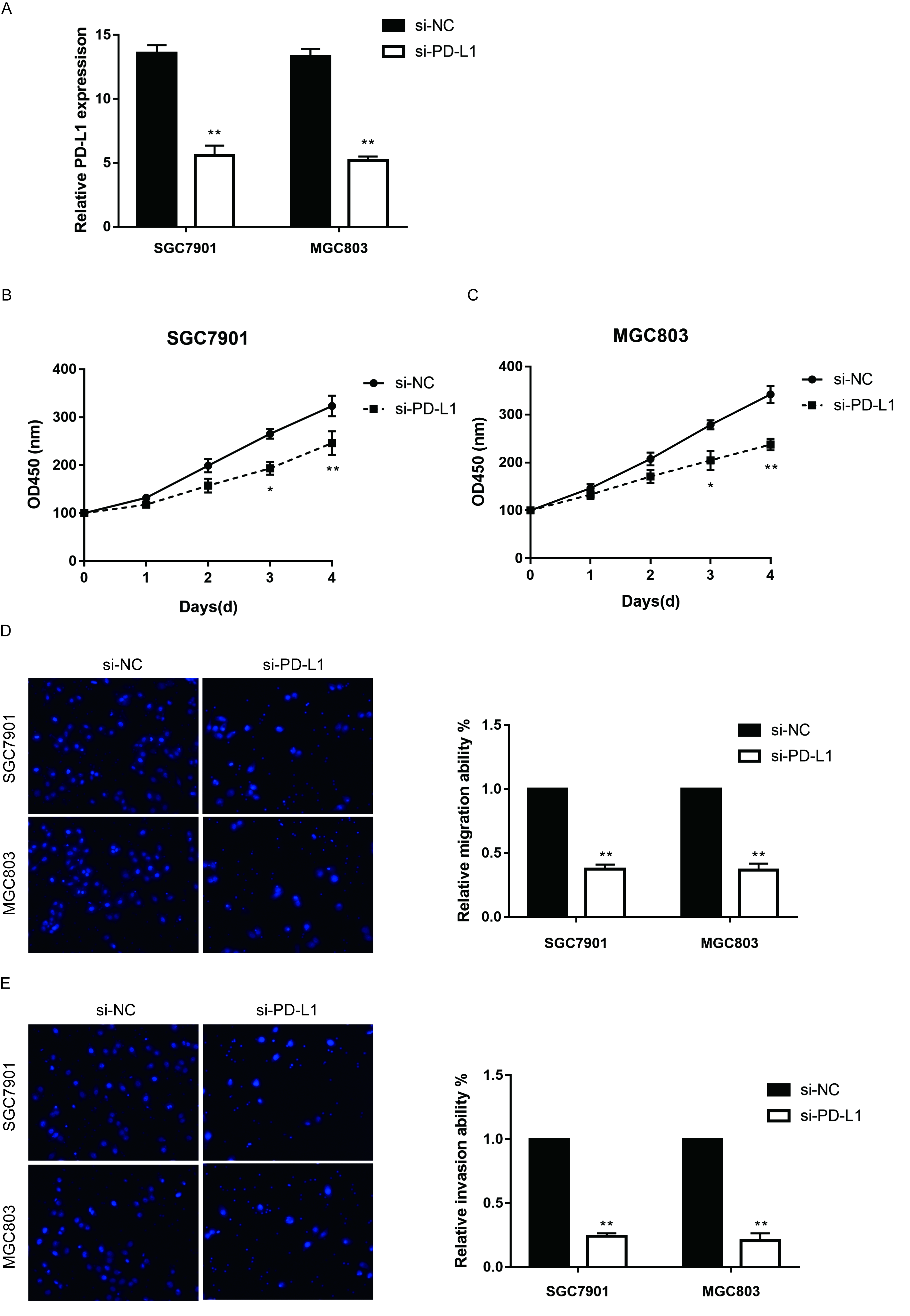

Supplement: Supplementary file 7 — Additional file 7: Figure S7. PD-L1 knockdown inhibits gastric cancer cells proliferation, migration and invasion. (A) The knockdown efficiency of si-PD-L1 was examined by qRT-PCR. (B and C) CCK8 assays of SGC7901 and MGC803 cells were performed after transfected with si-NC or si-PD-L1. (D and E) Transwell analysis of gastric cancer cells was performed after transfected with si-NC or si-PD-L1 in SGC7901 and MGC803 cells. Assays were performed in triplicate. **P < 0.01. [file 12935_2020_1479_MOESM7_ESM.tif]
